# Are safe guards at trampoline parks safe enough? A case report on a complete spinal cord injury after diving into a trampoline park foam pit

**DOI:** 10.1097/MD.0000000000018137

**Published:** 2019-11-27

**Authors:** Jae Eun Lee, Ju Hyun Kim, Chan Hee Park, Dae Won Gwak, Chul-Hyun Kim, Donghwi Park, Jong-Moon Hwang

**Affiliations:** a Department of Rehabilitation Medicine, Kyungpook National University Hospital; b Department of Rehabilitation Medicine, School of Medicine, Kyungpook National University; c Department of Rehabilitation Medicine, Daegu Fatima Hospital, Daegu, South Korea.

**Keywords:** complete spinal cord injury, foam cubes, safety measure, trampoline parks

## Abstract

**Rationale:**

Recently, commercial indoor trampoline parks have been opened around the globe, and both the number of venues and the park users are increasing. Academic literatures have largely focused on home trampoline related injuries, and less is known about the injuries associated with trampoline parks due to the limited number of studies or cases reported. In this report, we present a complete spinal cord injury sustained at a commercial indoor trampoline park.

**Patient Concerns:**

A 26-year old male developed tetraplegia after plainly jumping on the trampolines and diving into one of the foam pits head first.

**Diagnosis:**

C-spine CT revealed bilateral interfacetal dislocation on C6-7, and his C-spine MRI showed anterior translational injury at C6-7 with severe cord encroachment and complete discoligamentous complex disruption. He was diagnosed with complete spinal cord injury.

**Interventions:**

The patient underwent 30 minutes each of physical therapy and occupational therapy twice a day for a total of 25 days of in-patient rehabilitation. Interventions included tilt table, passive range of motion exercises, functional electrical stimulation, sitting balance training, upper extremity strengthening exercise, and hand manipulation exercises.

**Outcome:**

Despite intensive rehabilitation and the patient's good spirit, there was no functional change in all physical examinations between evaluations at initial and at discharge.

**Lessons:**

In conclusion, we aim to alert the risks associated with improper use of trampolines, promote safer entertainment environment, and aid in developing mandatory safety measures. We hope to alert the risks associated with improper use of trampolines, promote safer entertainment environment, and aid in developing mandatory safety measures.

## Abstract 2

Supplemental Digital Content is available in the text

## 1 Introduction

The popularity of trampolining as a form of recreation and exercise showed a rapid rise in the past few decades, and it became an official Olympic event in the 2000 Olympic Games.^[1,2]^ Recently, commercial indoor trampoline parks have been opened around the globe, and both the number of venues and the park users are increasing.^[3]^ There were only 25 trampoline parks in 2010 according to the International Association of Trampoline Parks, but the growth of industry has expanded to more than 350 trampoline parks in the world by 2014.^[4]^ As trampoline jumping poses a significant risk of injury, the rate of injuries raises due to the growth in popularity of the activity.^[5]^

Safety at trampoline parks is an emerging health concern, and there has been international attention drawn from the medical community as well as the media regarding the safety issues of such facilities.^[6]^ While most injuries related to trampoline use are minor and do not require hospitalization, severe injuries, as rare as they are, can be detrimental.^[2,6]^ Public health and preventive measures have focused mostly on domestic trampoline use.^[7]^ Despite the alarmingly increasing rates of preventable injuries associated with trampoline parks, current statements on the use of trampolines from societies such as the American Academy of Pediatrics and the Canadian Pediatric Society do not include consideration of commercial trampoline parks.^[2]^ Academic literatures have also largely focused on home trampoline related injuries, and less is known about the injuries associated with trampoline parks because of the limited number of studies or cases reported.

In the following case, we present a complete spinal cord injury sustained at a commercial indoor trampoline park. We hope to alert the risks associated with improper use of trampolines, promote safer utilization, and aid in developing future policies. This case was approved by the ethics committee of our hospital. And the patient signed an informed consent form for the publication of this case report.

## 2 Case report

A 26-year old male without past medical history visited an indoor trampoline park on March 10th, 2019. The trampoline park had wall-to-wall connected trampolines and was equipped with safe guards such as cushioned floors and walls. The trampolines were not raised off ground level but were built onto the ground, and the areas surrounding any jumping facility including frames and edges were padded to prevent severe injuries by falling from the trampolines. The park had constant supervision by park employees, and all users went through safety education before using the park facilities. The venue had foam pits which are the areas dedicated for performing stunts and jumping. The patient was plainly jumping on the trampolines and dived into 1 of the foam pits head first (Supplementary file 1). Although he did not perform any stunts such as somersaults or flips but simply dived into the foam pit, he obtained a complete loss of motor in his lower body immediately following the fall. The foam pit was approximately 65 cm deep from ground level and was filled with 14.5 cm × 14.5 cm sized tetrahedral foam cubes (Supplementary file 2, [Supplementary 2. Foam cube (sized 14.5 cm on each side) which filled a 65 cm deep pit foam, an area designated for jumping and diving]). Judging from the video clip of the injury, the assumed injury mechanism was hyperflexion of the patient's neck after his head got embedded in the cushioned foams. The rubbery material that the foams were made of is thought to have had significant friction coefficient which could have caused more flexion of the neck instead of letting the patient's head glide along the foams’ surface. The nature of the foam cubes is thought to have resulted in a more jarring stop, which would have increased the load on the patient's neck joints.

## 3 Clinical examination

On his initial physical examination, manual muscle test (MMT), Berg balance scale (BBS), modified barthel index (MBI), Jebsen-Taylor hand function test (JTHFT), and mini-mental state examination (MMSE) were checked. He showed normal motor in shoulder flexion, elbow flexion, and wrist extension. MMT in his elbow extensors marked F/F, finger flexors P/P, finger abductors P/P, and motor in his lower extremities were all zero. Sensory in his C6 dermatome and above was normal, but he complained of hypoesthesia in pain, temperature, and light touch in dermatomes C7 and below. Position, vibration sensation was absent in his lower extremities. More importantly, he had absent perianal sense, deep anal pressure, and showed zero anal contraction, which led us to diagnose him with complete spinal cord injury according to International standards for neurological classification of spinal cord injury.^[8]^ His initial BBS, MBI, JTHFT, and MMSE were 0, 5, 6/2, 30 respectively. Additionally, the patient suffered from significant neuropathic pain in all 4 extremities, needed a Foley catheter inserted for voiding, and also developed orthostatic hypotension. His initial Beck depression index (BDI) was 3, and the caregiver's BDI marked 15.

## 4 Studies and imaging

**Figure 1:**
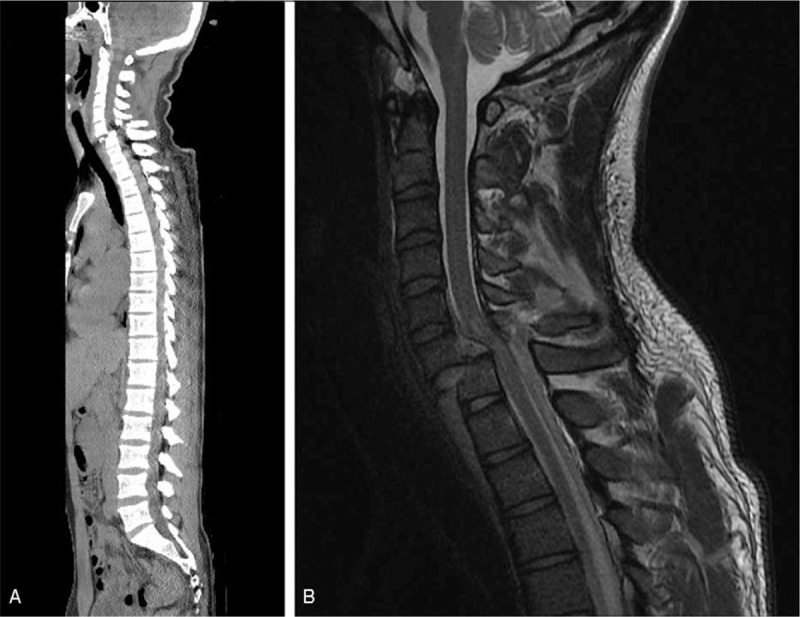
(A). Initial C-spine CT showing bilateral interfacetal dislocation on C6-7. (B) Initial C-spine MRI shows anterior translational injury at C6-7 with severe cord encroachment and complete discoligamentous complex disruption.

The patient was transferred to the emergency room of our hospital immediately. A C-spine CT revealed bilateral interfacetal dislocation on C6-7 (Fig. 1-A), and his C-spine MRI showed anterior translational injury at C6-7 with severe cord encroachment and complete discoligamentous complex disruption (Fig. 1-B).

## 5 Clinical course

He was admitted to the Neurosurgery Department and underwent posterior open reduction and pedicle screw fixation on C5-7 and anterior cervical discectomy and fusion on C6-7. After sufficient post operation care, he was referred to the Department of Physical Medicine and Rehabilitation on March 17th, 2019. We started physical therapy along with conventional treatments for his symptoms. Interventions included tilt table, passive range of motion exercises, functional electrical stimulation, sitting balance training, upper extremity strengthening exercise, and hand manipulation exercises. Thirty minutes each of physical therapy and occupational therapy were conducted twice a day during the weekdays for a total of 25 days of intensive rehabilitation therapy. The patient took 150 mg of Pregabalin twice a day for neuropathic pain and 10 mg of Escitalopram once a day for depressive mood changes that occurred as his quality of life deteriorated after the injury. Follow-up physical examinations did not show much improvement. The last follow-up before discharge was performed on April 10th, 2019, and while he showed improvement in neuropathic pain, numeric pain rating scale (NRS) decreasing from 3 to 1, his MMT remained similar, showing no significant change. His BBS and MBI were 1, 7 respectively. Urodynamic study which was performed before discharge revealed neurogenic bladder with low detrusor pressure and detrusor external sphincter dyssynergia. The patient failed to void and was transferred to another rehabilitation hospital with a Foley catheter inserted.

## 6 Discussion

A 26-year old male developed tetraplegia due to complete spinal cord injury after diving into a foam pit at a recreational indoor trampoline park. Simply diving into a foam pit designated for activities such as jumping and diving resulted in a debilitating injury that is likely to lead to long-term morbidity. He was diagnosed with complete spinal cord injury and underwent in-patient rehabilitation for 25 days. Despite intensive rehabilitation and the patient's good spirit, there was no functional change in all physical examinations between evaluations at initial and at discharge.

This case report highlights the alarming danger associated with commercial trampoline parks which are growing in popularity. Not enough is known regarding adequate safety measures in the parks setting.^[1,9]^ There is definite room for improvement in the park design and safety measures of indoor trampoline parks. Unexpected accidents occur in trampoline parks which seem acceptably safe, and both minor and major incidents should be reported so that evidence-based national safety standards can be developed. Standards regarding safe setup of the environment and trampolining activity itself are needed for the preventive measures. We cannot emphasize enough how crucial a mandatory guideline is needed.

Trampoline parks are designed and used in different ways comparing to home trampolines. Given that commercial trampoline parks are built for recreational purposes where users are more socially connected, behavioral change cannot be overlooked. The particular environment encourages risk taking and allows users to challenge their limits that would less likely occur elsewhere. Thus, the mechanism and nature of the injuries sustained at trampoline parks may differ from home trampoline use. In efforts to prevent injuries, strict safety instructions must be understood by all users and all safety guidelines should be respected when participating in trampolining.

An epidemiological study conducted by Schmitt and Gerner showed that the most common causation of spinal injury in trampolining and gymnastics is hyperflexion or sprain of the neck.^[10]^ The video clip of this case's injury suggests that the injury mechanism also was hyperflexion of the patient's neck. Another study by Silver obtained patient information including age, gender, level of cord injury, method of injury, and equipment failure in spinal injury cases that occurred during sports.^[11]^ Out of 150 patients, 121 had cervical spine injuries, and Silver explains that the cervical spine is particularly vulnerable because of its mobility and the disparity of the unsupported skull's movement on the cervical vertebrae. A flexion force exerted through the head to the cervical spine results in the crushing of the vertebral body and extrusion of the disc and vertebrae into the spinal cord. Bauze and Ardran revealed that when the vertex is locked on the ground as in this clinical case, far less force is needed to dislocate the cervical vertebrae and can occur without fracture.^[12]^

Ground level trampolines, padded edges and frames, cushioned walls and ground, supervision of park workers make trampoline parks seem a safe environment.^[7,13,14,15]^ Nevertheless, severe injuries including this case persist and raise the question, “Are the safe guards currently used at trampoline parks enough?” The foam pit where the patient sustained a detrimental injury was filled with 65 cm of cushiony foams. To our knowledge, no test institute has ascertained experimentally whether engineering interventions can improve the foam pit safety. Interventions such as installing a trampoline beneath the foam cubes or increasing the pit depth are some propositions that may improve safety, and experiment-based determinations are crucial before foam pit use in trampoline parks can be considered safe.

Follow-up time of the patient was not sufficient enough to determine the long-term outcome of the incidence, and no definite injury mechanism or risk factor can be drawn from this single case. However, some of the strengths of this case report include the available video clip of the accident and follow-up data of various behavioral data before and after in-patient rehabilitation. Through reporting a complete spinal cord injury caused by simple diving at an indoor trampoline park, we wish to raise attention regarding the danger risks of trampoline parks which are seemingly safe and are increasing in popularity, and to help provide information for future prevention measures.

## Author contributions

**Conceptualization:** Jae Eun Lee, Chul-Hyun Kim, Jong-Moon Hwang.

**Data curation:** Chan Hee Park, Dae Won Gwak.

**Investigation:** Ju Hyun Kim, Dae Won Gwak.

**Resources:** Ju Hyun Kim, Chan Hee Park.

**Supervision:** Chul-Hyun Kim, Donghwi Park, Jong-Moon Hwang.

**Writing – original draft:** Jae Eun Lee.

**Writing – review & editing:** Jae Eun Lee, Donghwi Park, Jong-Moon Hwang.

## Supplementary Material

Supplemental Digital Content

## Supplementary Material

Supplemental Digital Content
