# Supplementary material for: Are safe guards at trampoline parks safe enough? A case report on a complete spinal cord injury after diving into a trampoline park foam pit
Source: Medicine (Baltimore). 2019 Nov 27;98(48):e18137. doi: 10.1097/MD.0000000000018137 (PMC6890358; doi:10.1097/MD.0000000000018137)

**Supplementary 1.** Patient diving into a pit foam head first at a 40’ angle approximately. The dive resulted in complete spinal cord injury at the C4 level.


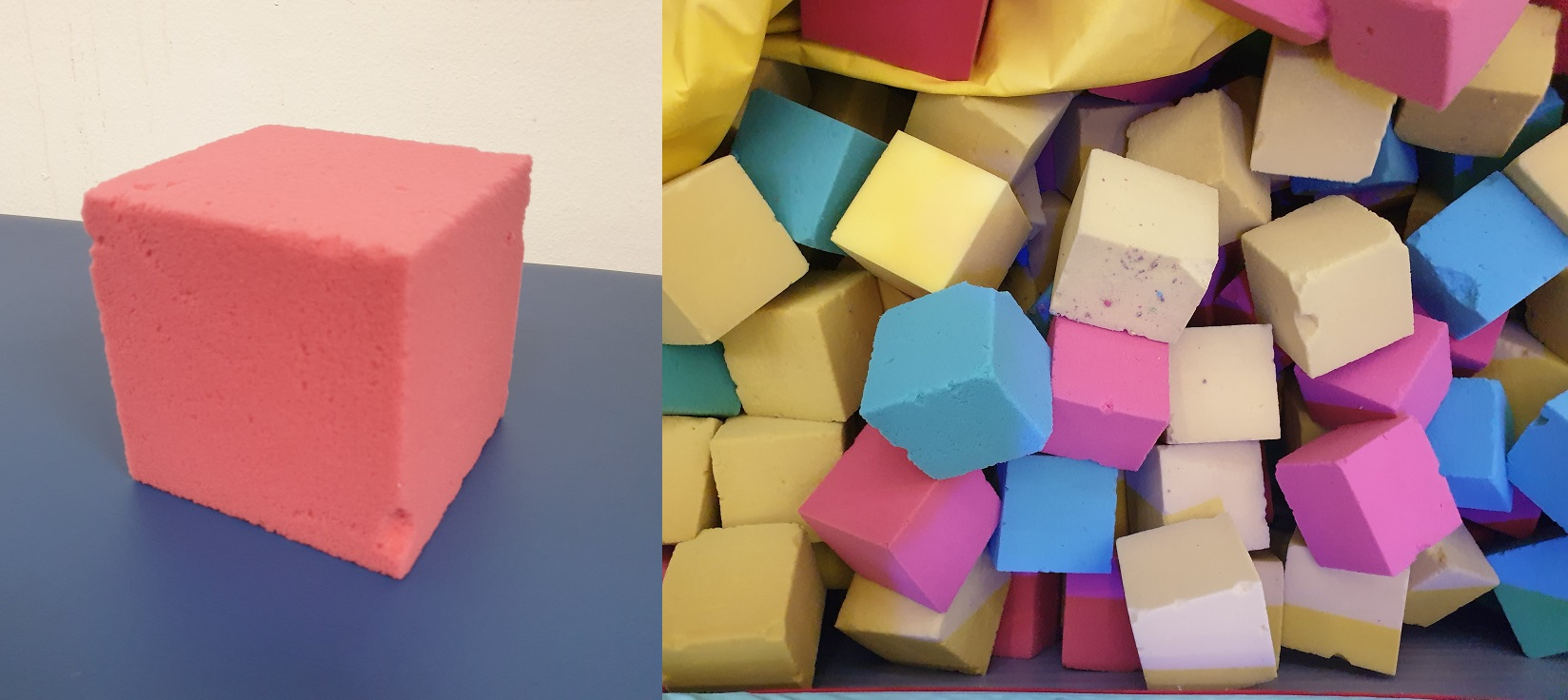

Supplement: Supplemental Digital Content [file medi-98-e18137-s001.doc]
